# No evidence for early fitness penalty in glyphosate‐resistant biotypes of *Conyza canadensis*: Common garden experiments in the absence of glyphosate

**DOI:** 10.1002/ece3.5741

**Published:** 2019-11-28

**Authors:** Zachery T. Beres, Micheal D. K. Owen, Allison A. Snow

**Affiliations:** ^1^ Department of Evolution, Ecology, and Organismal Biology Ohio State University Columbus OH USA; ^2^ Department of Agronomy Iowa State University Ames IA USA

**Keywords:** *Conyza canadensis*, fitness, glyphosate, herbicide, horseweed, resistance

## Abstract

Strong selection from herbicides has led to the rapid evolution of herbicide‐resistant weeds, greatly complicating weed management efforts worldwide. In particular, overreliance on glyphosate, the active ingredient in RoundUp^®^, has spurred the evolution of resistance to this herbicide in ≥40 species. Previously, we reported that *Conyza canadensis* (horseweed) has evolved extreme resistance to glyphosate, surviving at 40× the original 1× effective dosage. Here, we tested for underlying fitness effects of glyphosate resistance to better understand whether resistance could persist indefinitely in this self‐pollinating, annual weed. We sampled seeds from a single maternal plant (“biotype”) at each of 26 horseweed populations in Iowa, representing nine susceptible biotypes (S), eight with low‐level resistance (LR), and nine with extreme resistance (ER). In 2016 and 2017, we compared early growth rates and bolting dates of these biotypes in common garden experiments at two sites near Ames, Iowa. Nested ANOVAs showed that, as a group, ER biotypes attained similar or larger rosette size after 6 weeks compared to S or LR biotypes, which were similar to each other in size. Also, ER biotypes bolted 1–2 weeks earlier than S or LR biotypes. These fitness‐related traits also varied among biotypes within the same resistance category, and time to bolting was inversely correlated with rosette size across all biotypes. Disease symptoms affected 40% of all plants in 2016 and 78% in 2017, so we did not attempt to measure lifetime fecundity. In both years, the frequency of disease symptoms was greatest in S biotypes and similar in LR versus ER biotypes. Overall, our findings indicate there are no early growth penalty and possibly no lifetime fitness penalty associated with glyphosate resistance, including extremely strong resistance. We conclude that glyphosate resistance is likely to persist in horseweed populations, with or without continued selection pressure from exposure to glyphosate.

## Graphical abstract

Fitness effects related to varying levels of glyphosate resistance in *Conyza canadensis* were investigated over 2 years using common garden experiments in Iowa. Based on early growth measures, there appears to be no evidence for early fitness penalty in *C. canadensis* resistant to 40× glyphosate.

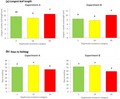

## 1 INTRODUCTION

The concomitant use of herbicides and genetically modified crops worldwide has altered the face of agriculture in recent decades. Increased exposure to a subset of available herbicides has spurred the rapid evolution of resistance in many weed species (Heap, 2019; Mortensen, Egan, Maxwell, Ryan, & Smith, 2012). Arguably, no herbicide has had a greater impact on modern agriculture than glyphosate (the active ingredient in RoundUp^®^), which has been called “a once‐in‐a‐century herbicide” and is currently the most widely applied herbicide worldwide (Benbrook, 2016; Duke & Powles, 2008). To date, at least 40 weed species have evolved resistance to glyphosate (Heap, 2019). This problem has led growers to use increased amounts of glyphosate and incorporate more toxic herbicides, such as 2,4‐D and dicamba, in weed management efforts (Benbrook, 2012, 2016).

The extent to which herbicide resistance spreads and persists in weed populations is heavily influenced by whether or not resistance mechanisms are associated with fitness effects in the absence of herbicide treatment (Bergelson & Purrington, 1996; Vila‐Aiub, Neve, & Roux, 2011). Thus, understanding fitness effects of resistance genes is important for developing weed management strategies and modeling approaches for mitigating the problem (Neve, 2008; Vila‐Aiub, Neve, & Powles, 2009b). Herbicide resistance may entail a fitness cost due to pleiotropic and other effects on plant growth and reproduction (Pedersen, Neve, Andreasen, & Powles, 2007; Vila‐Aiub, Neve, & Powles, 2009a; 2009b), although this is not always the case (Vila‐Aiub et al., 2014). When herbicide resistance does not confer a fitness cost, and may even be associated with a fitness benefit in the absence of an herbicide (Beres, Yang, et al., 2018; Wang et al., 2014), this trait has the potential to persist indefinitely in weed populations.

Unfortunately, many previous studies that have tested for fitness effects of herbicide resistance have not controlled for genetic background and often included comparisons between a single resistant population versus a single susceptible population. Vila‐Aiub et al. (2011), Vila‐Aiub, Gundel, and Preston (2015) and others have summarized best practices for this type of research and noted common deviations from best practices including: (a) comparisons based on only one or a few susceptible (S) and resistant (R) biotypes that could have differed in genetic background, (b) accessions represented by pooled samples from populations that could have included a mixture of S and R individuals, (c) resistance was not quantified beyond simple designations of S and R (ignoring possible variation in the degree of resistance), and/or (d) relatively small sample sizes that made statistical inferences difficult. A further challenge is that most studies, including the current study, measure components of fitness such as biomass or seed production, often referred to as fitness‐related traits, rather than using a full life‐cycle approach under realistic field conditions. Nonetheless, many earlier publications and those reporting only growth rates, biomass, or fecundity provide a starting point for understanding whether resistance traits are likely to persist after herbicide applications are discontinued.

Previous studies on the fitness effects of glyphosate resistance in agricultural weeds have reported either a fitness penalty or no fitness cost associated with resistance. Fitness costs have been found in some glyphosate‐resistant populations of *Kochia scoparia* in greenhouse studies, but not in others (Martin et al., 2017; Osipitan & Dille, 2017). Fitness‐related costs of glyphosate resistance also have been reported in glyphosate‐resistant populations of perennial ryegrass (*Lolium perenne* L.) and morning glory (*Ipomoea purpurea*; Yaniccari, Vila‐Aiub, Istilart, Acciaresi, & Castro, 2016; Debban, Okum, Pieper, Wilson, & Baucom, 2015). Another study by Wu, Davis, and Tranel (2017) found fitness costs associated with one mechanism of glyphosate resistance (EPSPS, EC2.5.1.19, gene amplification) but not for others in greenhouse‐grown, synthetic populations of *Amaranthus tuberculatus*. However, two well‐designed studies found no fitness costs associated with glyphosate resistance in Palmer amaranth (*Amaranthus palmeri*), a major problem weed in US agriculture (Giacomini, Westra, & Ward, 2014; Vila‐Aiub et al., 2014).

**Figure 1:**
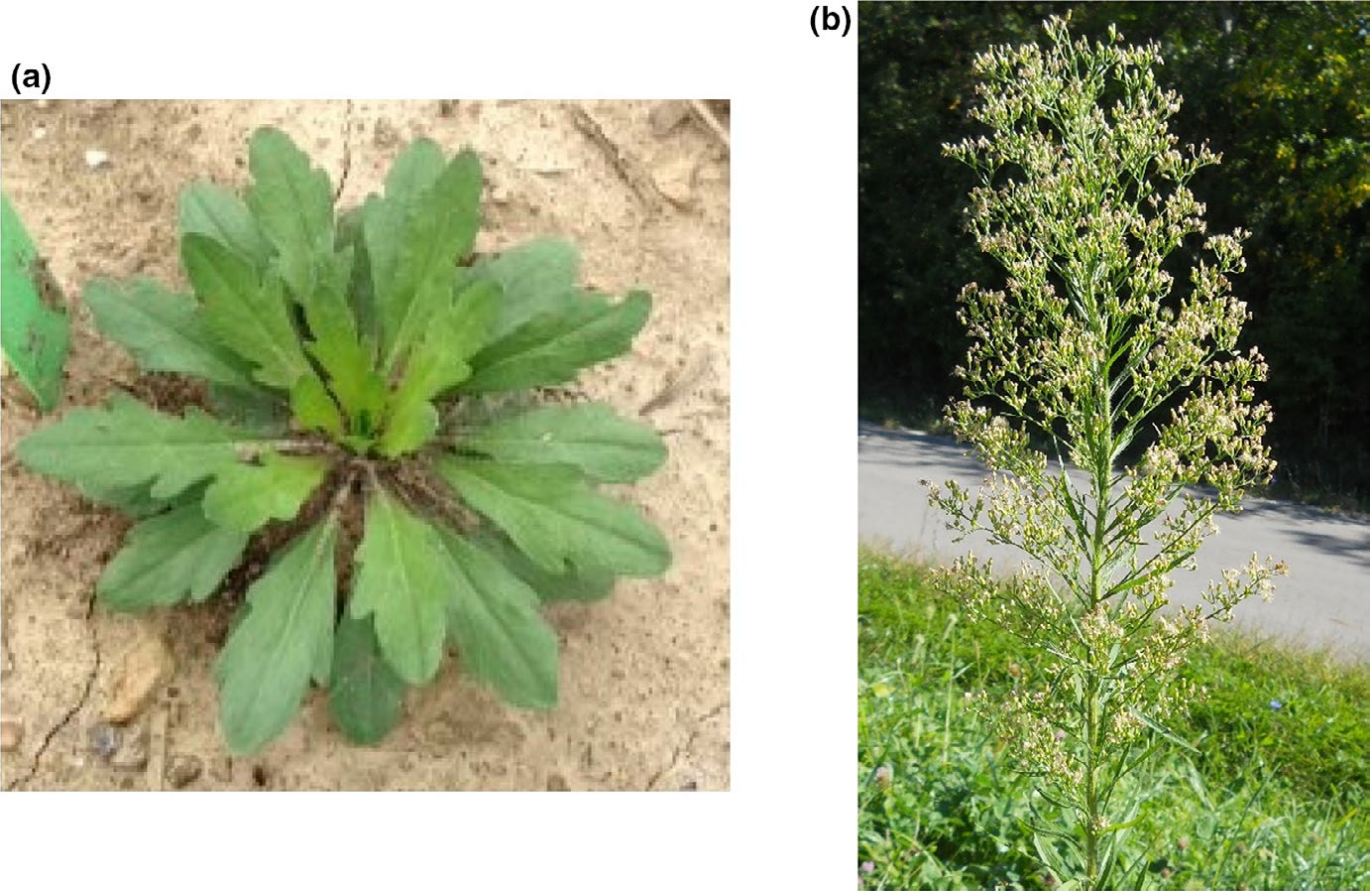
Representative photographs of *Conyza canadensis* as (a) rosette and (b) flowering

In the past several decades, glyphosate‐resistant horseweed (*Conyza canadensis*; Figure 1) has become an important weed problem in no‐tillage and low‐tillage crops, including soybean, vineyards, and orchards (Davis, Gibson, Bauman, Weller, & Johnson, 2009; Webster & Nichols, 2012). Horseweed can be managed by spring and fall tillage, and seeds fail to germinate when buried at depths greater than ~0.5 cm (Brown & Whitwell, 1988; Nandula, Eubank, Poston, Koger, & Reddy, 2006). However, after the commercialization of RoundUp Ready^®^ crops facilitated the increased adoption of no‐tillage crop production systems, horseweed became more abundant and more difficult to manage, especially after many populations evolved resistance to glyphosate (Beres, Ernst, et al., 2018; Davis et al., 2009; Davis, Gibson, & Johnson, 2008; Webster & Nichols, 2012). Uncontrolled or poorly managed horseweed populations can form nearly monospecific stands with high‐population densities and may reduce soybean yields by 90% (Bruce & Kells, 1990). Glyphosate‐resistant horseweed now occurs in at least 25 states in the United States and in 12 other countries (Heap, 2019). In Iowa, glyphosate‐resistant (GR) horseweed was first reported in 2011 (Heap, 2019), and some of the biotypes that we sampled in 2015 were able to survive when sprayed with 40 times the original manufacturer's recommended dosage (Beres, Ernst, et al., 2018). Several mechanisms for resistance have been reported in horseweed, including reduced translocation and vacuolar sequestration of the herbicide (Feng et al., 2004; Ge, d'Avignon, Ackerman, & Sammons, 2010; Koger & Reddy, 2005), increased production of EPSPS (Dinelli et al., 2006; Mei, Xu, Wang, Qiu, & Zheng, 2018; Tani, Chachalis, & Travlos, 2015), and a target‐site point mutation (Page et al., 2018; Beres et al., 2019). In California, multiple independent origins of glyphosate‐resistant horseweed have been documented using genetic markers (Okada et al., 2013).

Previous studies have tested for fitness costs in glyphosate‐resistant horseweed, but inferences from these studies are limited because they compared only one or two resistant (R) versus susceptible (S) populations, some of which may have included plants with varying levels of resistance. In California, a single resistant population had greater root biomass, greater total biomass, and/or faster growth than a single susceptible population (Alcorta, Fidelbus, Steenwerth, & Shrestha, 2011; Grantz, Shrestha, & Vu, 2008; Shrestha, Hanson, Fidelibus, & Alcorta, 2010; Shrestha, Hembree, & Va, 2007). Although it is not possible to generalize from single populations, these reports of fitness‐related *benefits*, rather than *costs*, are noteworthy, especially in the context of our findings reported here. Two other studies compared two susceptible versus two resistant populations; Davis et al. (2009) found negligible differences between S and R populations in terms of biomass, height, or seed production, while Gage et al. (2015) reported that one of two R populations produced significantly more seeds compared to other populations.

Unlike previous studies comparing S versus R horseweed biotypes at the level of populations, here we considered each maternal seed family to be a biotype, which we characterized as susceptible (S), low‐level resistant (LR), or extremely resistant (ER) to glyphosate, as described below. Because maternal biotypes are likely to represent full‐sib progeny due to self‐pollination (Okada et al., 2013; Smisek, 1995), genetic differences among biotypes are expected to be heritable and subject to selection following exposure to glyphosate. Thus, our approach provided a fine‐grained assessment of the extent of glyphosate resistance and associated phenotypic characteristics at the level of maternal seed families.

The main goals of our current study were to:

1. Compare fitness‐related traits of susceptible biotypes with those that were known to have low‐level versus extreme resistance to glyphosate.
2. Test for consistent differences in fitness‐related traits among biotypes in common garden experiments over two consecutive years.

## 2 MATERIALS AND METHODS

### 2.1 Study species

*Conzya canadensis* L. Cronq. (also known as horseweed, marestail, or Canada fleabane) was the first broadleaf weed species reported to evolve resistance to glyphosate and did so after only three years of repeated field applications in Delaware (VanGessel, 2001). Horseweed is a summer annual or facultative winter annual that is native to North America and has become prevalent worldwide (Weaver, 2001). This species is common in field margins, abandoned fields, roadsides, industrial areas, and other disturbed sites, in addition to row crops, orchards, vineyards, and other perennial crops (Dauer, Mortensen, & VanGessel, 2007; Hanson, Shrestha, & Shaner, 2009). Seed germination occurs whenever conditions are favorable (Buhler & Owen, 1997; Weaver, 2001). Rosettes bolt to produce a ~1–2 m tall, multibranched flowering stem (Figure 1; Regehr & Bazzaz, 1979; Weaver, 2001), and the small florets are highly self‐pollinating, with about 1%–4% outcrossing (Davis, Kruger, Hallett, Tranel, & Johnson, 2010; Zelaya, Owen, & VanGessel, 2004). Individual plants can produce >200,000 tiny, wind‐dispersed seeds that exhibit no dormancy and are relatively short‐lived in soil seed banks (Tozzi, Lyons, & Acker, 2014; Weaver, 2001). The seeds can disperse >500 km via the upper atmosphere (Shields, Dauer, VanGessel, & Neumann, 2006), but only ~1% of seeds disperse >100 m from their maternal plants (Dauer et al., 2007). Nonetheless, seeds from large, heavily infested fields could potentially disperse ~1–5 km per year (Dauer, Luschei, & Mortensen, 2009; Dauer et al., 2007).

### 2.2 Seed collections and biotype resistance

**Figure 2:**
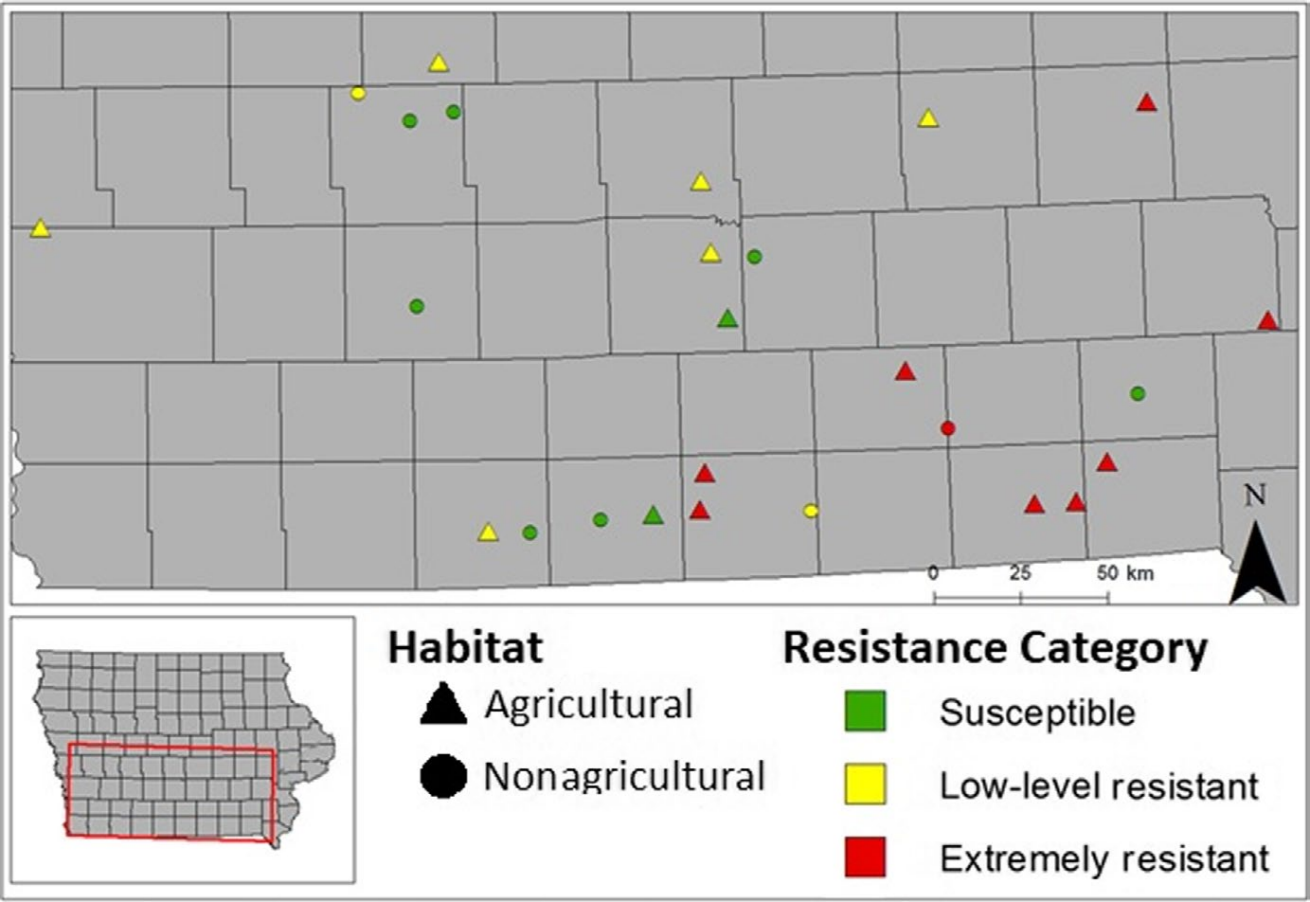
Collection site locations for 26 *Conyza canadensis* biotypes (maternal seed families) that were used in common garden experiments in southern Iowa, showing the glyphosate resistance category for each biotype. Triangles (▲) correspond to biotypes from soybean field habitats, and circles (●) correspond to those from nonagricultural habitats; further site details are listed in Table S1. Adapted from Figure 1 in Beres, Ernst, et al., 2018; maps were generated using ArcGIS^®^ software ver. 10.2.2 for Desktop (Environmental Systems Research Institute Inc., http://www.esri.com)

As noted above, we assumed that seeds from the same maternal plant were full sibs and we referred to these maternal seed families as individual biotypes. In a previous study, we confirmed that progeny from the same maternal plant had consistent levels of resistance to glyphosate (Beres, Ernst, et al., 2018). Here, we used common garden experiments to compare fitness‐related traits among a subset of the 74 biotypes described in Beres, Ernst, et al. (2018). Seeds were collected from one maternal plant per population from both agricultural and nonagricultural habitats in southern Iowa in 2015 (Figure 2). Glyphosate resistance for each biotype was characterized by spraying greenhouse‐grown rosettes with one of five dosages: 0×, 1× (840 g ae ha^−1^; manufacturer's recommended application rate, which equates to 0.6725% glyphosate (v/v); AquaMaster^®^, 648 g/L, Monsanto Co.), 8×, 20×, and 40× (see Beres, Ernst, et al., 2018 for details).

We selected 10 biotypes from each of three resistance categories: susceptible (S, <80% survival at 1×), low‐level resistant (LR, at least 80% survival at 1× but <80% survival at 8×), and extremely resistant (ER, >80% survival at 40×) for the common garden experiments. Due to limited seed supply and unequal germination, the final numbers of biotypes used in the current study were 9 S, 8 LR, and 9 ER, each sampled from a different location (Figure 2). We attempted to include a broad geographic range for each category, but most of the ER biotypes were from populations in southeastern Iowa, where no‐till soybean production is common, while the S and LR biotypes were more evenly dispersed. Seven of the S biotypes were collected from nonagricultural habitats, while only two LR biotypes and one ER biotype came from nonagricultural habitats (Figure 2). The agricultural habitats were soybean fields, most of which were likely sprayed with glyphosate prior to seed collection (Beres, Ernst, et al., 2018). GPS coordinates of the collection sites are listed in Table S1.

### 2.3 Common garden experiments

To compare fitness‐related traits of S, LR, and ER biotypes in the absence of glyphosate, we conducted two common garden experiments, Experiment A and Experiment B, in the summers of 2016 and 2017, respectively. In each year, we used the same two field sites near Ames, Iowa, Site 1 (42.0040 N, 93.3957 W) and Site 2 (41.5847 N, 93.4151 W). Both sites were tilled prior to beginning the experiments, and no fertilizers or herbicides were used at either site for the duration of the experiments.

For each experiment, we used a randomized, complete block design in which 25 initial greenhouse trays and corresponding designated rows in each field were considered as blocks (i.e., plants in the same tray were used in the same row). Thus, the design for each year included 2 field sites × 25 blocks per site × 26 biotypes, for a total of 1,300 plants per year. Seeds for each site were germinated one week apart, with Site 1 seeds planted first. Seeds were germinated in 3″ round, biodegradable Jiffy pots (BFG Supply; http://www.bfgsupply.com) filled with moistened Fafard #2 soil (http://www.fafard.com) and were thinned to one seedling per pot one week after planting. Pots were randomly positioned within trays, with one plant per biotype in each tray. Trays were watered as needed and rotated weekly to mitigate effects of any environmental variation in the greenhouse. The greenhouse was maintained at 18‐21/23‐26 C (night/day), and supplemental lights (400‐watt metal halide) were used for 14 hr per day. After 3 weeks in the greenhouse, trays were moved outdoors to acclimate the plants to full sunlight for 3 days prior to transplanting into common gardens.

To avoid variation due to competition with other plants, each field site was covered with Heavyweight Woven Pro‐5 Weed Barrier fabric (Gempler's; http://www.gemplers.com), with holes cut for the potted plants every 1.2 m along each of 25 rows. Each row had one plant per biotype randomly assigned a position in the row. Transplanting was staggered between Site 1 and Site 2 by 8 days (2016) or 10 days (2017) during May. During transplanting, the plants were prewatered and the outer edge of each peat pot was removed to allow direct soil‐to‐soil contact in the ground. After transplanting, each plant was hand‐watered again, and watering continued for the first two weeks as needed. Sickly or dead plants were removed and replaced with healthy alternates during the first week after transplanting to account for transplant shock (a total of six transplants were replaced in 2016 and four in 2017).

After transplanting, all plants were monitored weekly to record survival and disease symptoms. More than 99% of the plants survived to bolting, but a large portion developed disease symptoms in both years (40% in 2016 and 78% in 2017), as described further below. Therefore, we focused our analyses on early growth and days to bolting for both years. We measured the length of the longest rosette leaf to characterize relative differences in rosette size among biotypes over time. Leaf length measurements were taken at 6 weeks after transplant (WAT). We also recorded the number of days from transplanting to the onset of bolting for each plant. A plant was recorded as bolting when the stem had grown at least 2 cm above the basal rosette.

In 2017, we measured the dry above‐ground biomass of nondiseased plants that were harvested at the end of the growing season in mid‐October. These measurements were used in a regression analysis to determine the degree to which rosette size at 6 WAT was correlated with the end‐of‐season biomass of nondiseased plants (for all biotypes combined due to small sample sizes within biotypes).

### 2.4 Statistical analyses

#### 2.4.1 ANOVAs

For rosette size at 6 WAT and the number of days to bolting, significant differences among biotypes at *p* < .05 were inferred using ANOVAs and Tukey's multiple comparisons. No data transformations were needed to meet assumptions of normality or homogeneity of variance. Each experiment (i.e., each year) was analyzed separately to simplify the models and focus on within‐year effects.

For each experiment, we ran two statistical models: one to compare biotypes nested within the three resistance categories (S, LR, and ER), and the other to compare all biotypes with each other. Both models began as full models using all terms and interactions, and we dropped nonsignificant terms until we had the reduced models presented here. In the first model (PROC GLIMMIX, SAS version 9.4; SAS Institute Inc., 2019), we treated the effects of field site, row (nested within field site), and biotype (nested within glyphosate resistance categories) as random variables, while glyphosate resistance category was considered fixed. For this general linear mixed model, we did not necessarily care about the variation among the biotypes, but rather wanted to test for differences among glyphosate resistance categories (S, LR, ER). Each random factor was tested with a log‐likelihood ratio test to determine whether its variation was significantly different from zero (Bolker et al., 2009). Because horseweed rosettes are circular, we also explored using the longest leaf length as the radius to estimate rosette area (=π*r* ^2^). However, for this analysis and others, using estimated rosette area instead of longest leaf length did not change the outcome of any statistical comparisons, so only leaf length data are summarized here.

In the second model (PROC GLM, SAS version 9.4; SAS Institute Inc., 2019), we specifically wanted to test for differences among the biotypes. Here, we treated field site, row, and position within rows as random variables while biotype was considered fixed. This model also included two interaction terms: one between field and row, and another between field and biotype. We considered biotype as a fixed factor here because we are interested in the variation that exists among these particular biotypes, independent of their glyphosate resistance category.

#### 2.4.2 Regression analyses

We were interested in the relationship between the rosette size at 6 weeks after transplanting and the number of days to bolting because bolting marks the transition from vegetative to reproductive growth and may be related to plant size. An exponential line of best fit was fit to the data for Experiment A and Experiment B. The relationship between the mean number of days to bolting and mean longest rosette leaf length at 6 WAT was inferred using ANOVA (PROC GLM, SAS version 9.4, SAS Institute Inc., 2019).

We also wanted to characterize the relationship between rosette size at 6 weeks after transplanting and final above‐ground dry biomass to determine the extent to which rosette size was correlated with final biomass. Due to the prevalence of diseased plants late in the growing season, a subset of phenotypically “normal” plants (i.e., those that exhibited no disease symptoms postbolting) from Experiment B was selected. A linear line of best fit was fit to the data. The relationship between rosette size at 6 WAT and dry, above‐ground biomass was inferred using ANOVA (PROC GLM, SAS version 9.4; SAS Institute Inc., 2019).

#### 2.4.3 Frequency of disease symptoms

Disease symptoms were monitored throughout each growing season. Within each glyphosate resistance category and year, the final percentage of plants with disease symptoms was calculated. A chi‐square test of independence followed by pairwise comparisons with a Bonferroni correction was used to determine significant differences in percent diseased among the three resistance categories for each experiment.

## 3 RESULTS

### 3.1 Longest rosette leaf length

We used the length of the longest rosette leaf (hereafter, “longest leaf length”) as a nondestructive measure of plant size prior to bolting. Leaf length at 6 weeks after transplanting (WAT) was positively correlated with final above‐ground dry biomass of nondiseased plants (Experiment B: *R* ^2^ = 0.46, *p* < .0001, *N* = 314). Because of the prevalence of disease later in the growing season, we focus on rosette size prior to bolting as a proxy for lifetime fitness.

**Figure 3:**
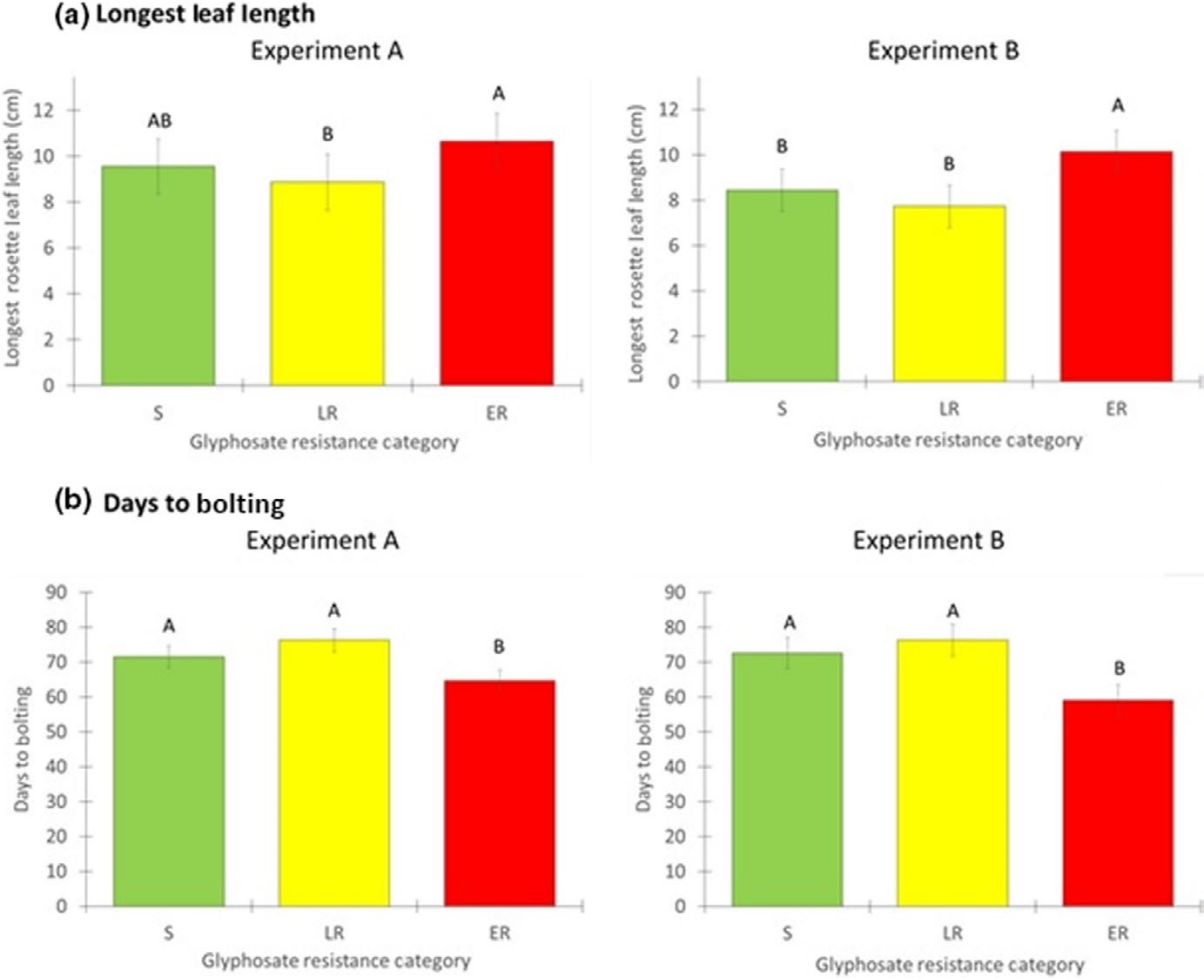
Differences among glyphosate resistance categories in (a) the longest rosette leaf at 6 weeks after transplanting and (b) the number of days from transplanting to bolting in Experiments A and B. Resistance categories are S (susceptible), LR (low‐level resistant), and ER (extremely resistant). Data from both sites in each year are combined, and least‐squares means + 1 *SE* are shown; *N* (plants per resistance category) = 445 S, 384 LR, and 439 ER for Experiment A; *N* = 437 S, 349 LR, and 447 ER for Experiment B. In each comparison, means with different superscripts are significantly different at *p* ≤ .05 (Tukey's tests following nested ANOVAs)

Analysis from the nested ANOVA model applied to longest leaf lengths at 6 WAT showed significant differences among the three resistance categories (Table 1, Figure 3a, b). In Experiment A, ER biotypes had significantly longer leaves than LR, while S biotypes were intermediate (mean lengths were 10.7, 8.7, and 9.6 cm, respectively). In Experiment B, leaves of ER biotypes were significantly longer than both LR and S biotypes, which did not differ from each other (mean lengths of 10.1, 7.9, and 8.4 cm, respectively). Thus, these results were relatively consistent in both years, with ER biotypes attaining relatively larger rosette sizes, especially compared to the LR biotypes.

**Table 1:** Nested ANOVAs for main effects of glyphosate resistance categories on the length of the longest rosette leaf at six weeks after transplanting in Experiment A (2016) and Experiment B (2017)

Experiment A				
Random effects	*df*	*−2Res log like	*X* ^2^	*p*
Field	1	5,560	33.1	<.0001
Row (field)	1	5,560	33.1	<.0001
Biotype (category)	1	5,708	180.7	<.0001
Fixed effects	*df* (num, den)	*F*	*p*	
Category	2, 23	5.61	.0104	
Experiment B				
Random effects	*df*	*−2Res log like	*X* ^2^	*p*
Field	1	5,059	30.2	<.0001
Row (field)	1	5,059	30.2	<.0001
Biotype (category)	1	5,160	130.9	<.0001
Fixed effects	*df* (num, den)	*F*	*p*	
Category	2, 23	10.38	.0006	

Note

Rows are nested within fields; biotypes are nested within categories. See Figure 3 for category means and Tukey's test comparisons.

**Figure 4:**
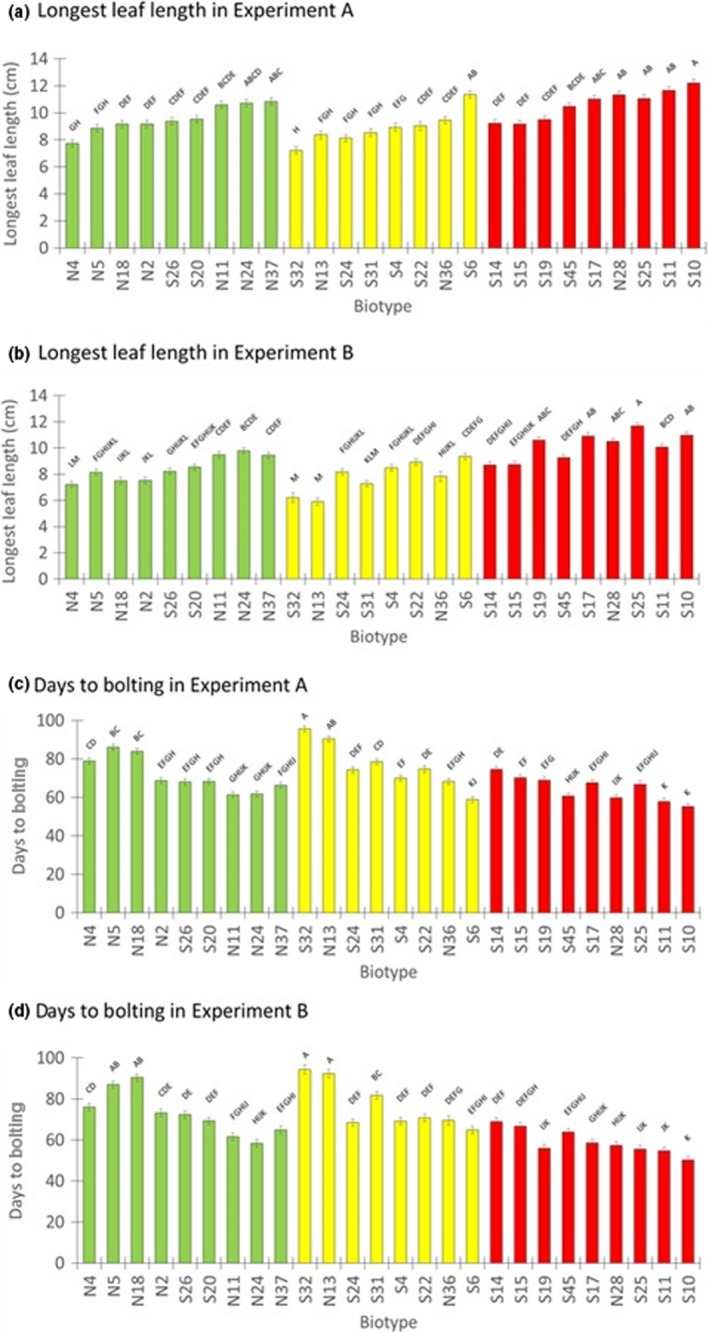
Differences among the 26 horseweed biotypes in length of the longest rosette leaf at 6 weeks after transplanting and days to bolting in Experiment A (a, c) and Experiment B (b, d). Biotypes are denoted as S for susceptible (green bars), LR for low‐level resistant (yellow bars), and ER for extremely resistant (red bars). Biotype ID labels beginning with “S” are from agricultural habitats, and those beginning with “N” are from nonagricultural habitats. Least‐squares means ± 1 *SE* are shown, with data from both sites in each year combined; *N* = 48–50 plants per biotype except for six means with *N* = 32–46 (see Table S1). Means that do not share superscripts are significantly different at *p* ≤ .05 (Tukey's tests)

Analysis of longest leaf lengths from the second, non‐nested ANOVA model showed significant differences among the 26 biotypes, including differences among biotypes within each resistance category (Figure 4a, b.). At 6 WAT, the mean longest leaf length for each biotype ranged from 7.3 to 12.2 cm in Experiment A and 6.0 to 11.7 cm in Experiment B (Figure 4a, b). In general, relative differences among biotypes were similar in both years. For example, biotypes S32 and N13 were relatively small in both years, whereas S25, S11, and S10 were relatively large among the group of 26 biotypes. Across all 26 biotypes, however, many biotypes were not significantly different from one another despite being from different resistance categories, reflecting a large amount of overlap in the performance of S, LR, and ER biotypes.

### 3.2 Time to bolting

More than 99% of the plants in both experiments survived to bolting, which began about 7 weeks after transplanting. Analyses of bolting time using the nested ANOVA model showed significant and consistent differences among the three resistance categories (Table 2, Figure 3). In both Experiment A and Experiment B, ER biotypes bolted significantly earlier than both S and LR biotypes, which were not significantly different from each other. In Experiment A, ER biotypes bolted 6.9 days earlier on average than S biotypes and 11.7 days earlier than LR biotypes (Figure 3b). Similarly, in Experiment B, ER biotypes bolted 13.4 days earlier on average than S biotypes and 17.2 days earlier than LR biotypes (Figure 3b).

**Table 2:** Nested ANOVAs for main effects of resistance categories on the number of days to bolting in Experiment A (2016) and Experiment B (2017)

Experiment A				
Random effects:	*df*	*−2Res log like	*X* ^2^	*p*
Row (field)	1	9,714	11.0	.0009
Biotype (category)	1	10,245	542.1	<.0001
Fixed effects:	*df* (num, den)	*F*	*p*	
Category	2, 23	3.36	.052	
Experiment B				
Random Effects:	*df*	*−2Res log like	*X* ^2^	*p*
Field	1	9,792.02	13.0	.0003
Row (field)	1	9,792.02	13.0	.0003
Biotype (category)	1	9,836.19	57.2	<.0001
Fixed effects:	*df* (num, den)	*F*	*p*	
Category	2, 23	7.62	.0029	

Note

Rows are nested within fields; biotypes are nested within categories. See Figure 3 for category means and Tukey's test comparisons.

Across all 26 biotypes, the earliest biotype to bolt was S10 (means of 55.1 and 50.0 days in Experiments A and B, respectively; Figure 4c, d), while the latest was S32 (means of 95.6 and 93.1 days, respectively). Mean bolting times for each biotype were consistent across years and differed significantly among some of the biotypes in the same resistance categories based on the non‐nested ANOVA model (Figure 4c, d). Regardless of resistance categories, biotypes that bolted earliest were those that had the longest leaf lengths at 6 WAT (Figure 4). For example, S6 (LR) and S10 (ER) were among the largest rosettes at 6 WAT and were among the first to bolt. Exponential regressions for mean bolting date as a function of mean longest leaf length had *R* ^2^ values of 0.76 for Experiment A and 0.86 for Experiment B (*N* = 26 biotypes; *y* = 174.63e^−0.094^ *^x^* and *y* = 171.11e^−0.105^ *^x^*, respectively).

### 3.3 Frequency of disease symptoms

**Figure 5:**
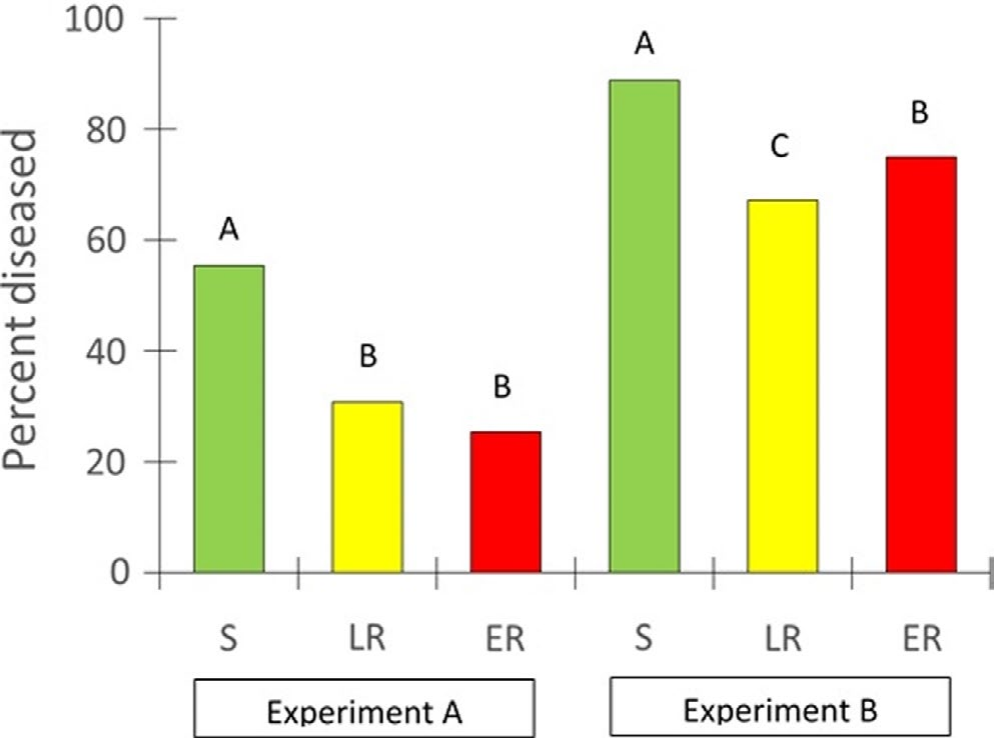
Percent of plants with disease symptoms in each glyphosate resistance category for Experiment A (2016) and Experiment B (2017). Resistance categories are S (susceptible), LR (low‐level resistant), and ER (extremely resistant). Data from both sites in each year are combined; *N* (plants per resistance category) = 445 S, 384 LR, and 439 ER for Experiment A; *N* = 437 S, 349 LR, and 447 ER for Experiment B. Within each experiment, means that do not share superscripts are significantly different at *p* ≤ .05. See Table S1 for percent diseased for each of the 26 biotypes in Experiments A and B

After bolting, disease symptoms were common, representing 40% of all plants in Experiment A and 78% of all plants in Experiment B (Figure 5). The most common symptoms included thickened ribboning of the stem, irregularly branched flower heads, often in stunted clusters, and/or partial or complete sudden necrosis of stems and leaves, similar to symptoms of a mycoplasma disease known as aster yellows (Regehr & Bazzaz, 1979). In each year, disease levels were similar at the two field sites (data not shown). Combining data for each resistance category, we found that the frequency of disease symptoms in Experiment A was ~2× greater for S plants compared to LR and ER plants, which were similar to each other (Figure 5). A similar pattern was seen in Experiment B, when many more plants were diseased, and differences between S plants and the two resistant categories were not as pronounced. Frequencies of disease symptoms were highly variable among biotypes and were not consistent for each biotype between years (Table S1).

## 4 DISCUSSION

Understanding underlying fitness effects associated with herbicide resistance is useful for recommending weed management strategies and anticipating whether such traits could persist indefinitely (Vila‐Aiub et al., 2014). Here, we found no evidence for early fitness costs of glyphosate resistance in *Conyza canadensis*, a major weed of low‐till agricultural crops. Instead, we found several indications of potential fitness benefits. As discussed further below, the ER biotypes consistently grew as well if not better than the low‐level resistant and susceptible biotypes in both experiments. Also, the ER and LR biotypes were less likely to exhibit disease symptoms compared to susceptible biotypes in both experiments.

### 4.1 Rosette size and days to bolting

By including two levels of glyphosate resistance, low‐level versus extreme resistance, we were able to identify interesting differences between these two resistance categories in fitness‐related traits. Our findings were somewhat counter‐intuitive in that the extremely resistant (ER) biotypes grew as well or better than those that were less resistant. As a group, LR biotypes were similar to S biotypes in rosette size and the number of days to bolting, while ER biotypes were about 12%–20% larger in rosette diameter at 6 WAT, and bolted 1–2 weeks earlier compared to S biotypes. If greater rosette size is correlated with greater lifetime fecundity, this trait can be considered as a component of overall fitness. We found a positive correlation between rosette size and end‐of‐season biomass in nondiseased plants, and previous studies of horseweed showed that both biomass and height of the flowering shoot are positively correlated with seed production per plant (Regehr & Bazzaz, 1979; Shrestha et al., 2010). Therefore, we expect that biotypes with larger rosettes would have produced more seeds per plant, had we been able to measure this.

We also found a strong correlation between large rosette size and earlier bolting across all 26 biotypes, suggesting a causal connection between these traits, similar to studies of other annuals such as Indian tobacco (*Lobelia inflata)* and smooth rockcress (*Arabis laevigata* var. *laevigata*; Bloom, Baskin, & Baskin, 2003; Simons & Johnston, 2003). Earlier flowering could lead to greater fecundity if later flowering is associated with deteriorating growing conditions, greater herbivore pressure, greater disease pressure, and/or a competitive disadvantage compared to neighboring plants (Snow & Stanton, 1988). Davis et al. (2008) found that horseweed plants with flowering shoots that emerged above the leaf canopy in soybean fields had better survival and seed production than those that remained below the canopy. Emergence above the soybean canopy could be influenced by both the timing of seedling emergence and the growth rates of young plants.

Previous studies involving comparisons between S and R biotypes of horseweed are consistent with our findings. In California, a series of studies with the same two populations, an S population from Fresno County and an R population from Tulare County, found that the R biotypes flowered earlier, were more competitive, attained greater biomass, and produced more seeds per plant than the S biotype (Alcorta et al., 2011; Grantz et al., 2008; Shrestha et al., 2010, 2007). Other studies with relatively low statistical power reported no clear differences between R and S biotypes in these traits (Davis et al., 2009; Gage et al., 2015; Zelaya et al., 2004). In a close relative, *Conyza bonariensis*, Travlos and Chachalis (2013) found no significant difference in the competitive ability of plants from one R population versus one S population in a deWit replacement series experiment. An advantage of the current study is that we used field‐grown maternal lines from 8 to 9 populations in each resistance category to examine heritable variation in fitness‐related traits.

### 4.2 Disease prevalence

Horseweed is known to be susceptible to several fungal and microbial diseases, a common one being aster yellows (Weaver, 2001). Aster yellows is caused by a mycoplasma transmitted by the aster leaf hopper, *Macrosteles fascifrons* Stal. (Regehr & Bazzaz, 1979). Disease symptoms that affected a large portion of the plants in our common garden experiments were similar to those reported for aster yellows by Regehr and Bazzaz (1979). They monitored naturally seeded populations of horseweed in Illinois and estimated that 80% of the plants were infected by aster yellows, resulting in a 53% reduction in seed production. We observed similarly high frequencies of disease symptoms in pilot field experiments with susceptible and resistant biotypes in Ohio (unpublished data).

In the current study, frequencies of disease symptoms increased from 40% of all plants in 2016 to 78% in 2017 (Figure 5). In both years, S biotypes were significantly more likely to have disease symptoms that LR or ER biotypes, which were generally similar to each other (Figure 5). It does not appear that slower growth or later bolting contributed to greater infection rates in S biotypes because they were similar to LR biotypes in terms of size and bolting times. In any case, we conclude that glyphosate resistance was not associated with a disease‐related fitness cost, and may even be associated with a fitness benefit in terms of disease symptoms.

### 4.3 Limitations

Several aspects of our study should be considered when drawing general conclusions about possible fitness effects of glyphosate resistance in *C. canadensis*. One caveat is that if the 8–9 biotypes that we sampled are not representative of their respective resistance categories, significant differences among categories in the traits we examined could be due to other factors, perhaps related to their sites of origin. For example, these biotypes were collected from a mixture of agricultural and nonagricultural sites (Figure 2). Most of the LR and ER biotypes occurred in soybean fields and may have been exposed to glyphosate earlier in the season, but several were collected from nonagricultural sites (Figures 2 and 4). Because horseweed has wind‐dispersed seeds capable of long‐distance movement, we assume that seeds can disperse between agricultural and nonagricultural sites, thereby limiting the potential for local adaptation to these contrasting habitats. In terms of the traits we examined, no clear patterns were seen in comparisons between biotypes from agricultural versus nonagricultural sites (Figure 4). Also, all but one ER biotype originated from the southeastern portion of the sampled area, where low‐till soybean production involving RoundUp Ready^®^ soybean seed sources is most common. However, because the nested ANOVAs detected significant differences among the three resistance categories, and because similar results were obtained in Experiments A and B, we suggest that such differences are robust for the biotypes included in this study.

Another caveat regarding our common garden experiments, which were planted in May, is that horseweed can be a summer or winter annual (Weaver, 2001). Both spring and autumn germination peaks can occur in the same populations, and the relative importance of these cohorts to overall population‐level seed production is thought to depend on environmental conditions for germination, winter survival, and the timing of tillage in agricultural fields (Buhler & Owen, 1997; Davis et al., 2008; Regehr & Bazzaz, 1979). We do not know whether the relative differences among glyphosate resistance categories that we observed in rosette size, days to bolting, and disease levels would be similar for plants that germinate in the autumn. Furthermore, the planting dates of our spring‐germinated plants overlapped with naturally occurring local plants, but their rosettes took longer to bolt and flower than what we observed in local populations (as in Davis et al., 2009). This delay could be related to the lack of competition imposed by using weed‐block fabric around each plant. Future experiments could more closely mimic naturally occurring horseweed populations, but we suspect that the general findings from our study would be confirmed.

## 5 CONCLUSIONS

Like many other weed species, horseweed has evolved several mechanisms for glyphosate resistance and the fitness effects associated with specific mechanisms have not been studied to date. In Beres et al. (2019), we report that ER biotypes from Iowa and Ohio have a point mutation at p185 of EPSPS2. This target‐site mutation has not been reported previously in horseweed populations in the United States (Nol, Tsikou, Eid, Livieratos, & Giannopolitis, 2012) or China (Mei et al., 2018), but it is known to occur in Ontario, Canada, where GR horseweed was first documented in 2010 (Page et al., 2018). We hypothesize, and the latest review by Vila‐Aiub, Yu, and Powles (2019) suggests, that different mechanisms of glyphosate resistance, such as point mutations, EPSPS gene amplification, and vacuolar sequestration, may have different fitness effects in the absence of glyphosate in horseweed and other species. In the current study, we did not attempt to identify specific resistance mechanisms in the sampled horseweed biotypes and this would be a fruitful avenue for further research.

In any case, it is clear that current ER horseweed biotypes do not appear to suffer any early fitness costs, and some may exhibit fitness benefits in terms of having larger rosettes, earlier bolting, and less susceptibility to pathogens compared to S biotypes. We assume that the selective pressures leading to the evolution of these traits are strongest in agricultural fields where glyphosate applications are common and surviving biotypes have high fecundity. Seed dispersal then distributes these biotypes across the landscape, where they also colonized nonagricultural habitats. In a previous study, for example, we detected ER biotypes in 9% of nonagricultural sites in Iowa and 62% in Ohio (*N* = 33 and 43, respectively; Beres, Ernst, et al., 2018).

In summary, the widespread and repeated evolution of herbicide resistance in weed species represents a textbook example of rapid evolutionary adaptation in the face of strong selection pressures. Previous studies suggest that glyphosate resistance can evolve repeatedly (Okada et al., 2013) and fairly quickly (VanGessel, 2001) in horseweed. Mounting evidence from this species and others indicates that fitness costs associated with resistance to glyphosate and other herbicides can be negligible, in which case overreliance on these herbicides has left a lasting evolutionary imprint on the gene pools of resistant weed populations. When this occurs in weeds that are capable of severely reducing crop yields, as with horseweed, growers must rely on alternative management strategies such as spring tillage, crop rotation, herbicide rotation, and/or cover crops to suppress weed populations.

## CONFLICT OF INTEREST

The authors declare that they have no competing interests.

## AUTHOR CONTRIBUTIONS

AAS, ZTB, and MDKO conceptualized the study. ZTB curated the data. ZTB involved in formal analysis. AAS and MDKO acquired funding. ZTB investigated the study. AAS, ZTB, and MDKO contributed to the methodology. AAS administrated the project. AAS and MDKO provided the resources. ZTB contributed software. AAS and MDKO supervised the study. ZTB validated the data. AAS and ZTB visualized the data. ZTB, AAS, and MDKO wrote the original draft of the manuscript. ZTB, AAS, and MDKO reviewed and edited the manuscript.

## Data Availability

Data for this study are available at https://DataDryad.org: https://doi.org/10.5061/dryad.fj6q573q1.
