# Supplementary material for: No evidence for early fitness penalty in glyphosate‐resistant biotypes of Conyza canadensis: Common garden experiments in the absence of glyphosate
Source: Ecol Evol. 2019 Nov 28;9(24):13678–89. doi: 10.1002/ece3.5741 (PMC6953693; doi:10.1002/ece3.5741)
Supplement: Supplementary file 1 [file ECE3-9-13678-s001.docx]

Supplemental Table 1. Locations of sampled biotypes, habitat types, and percent of plants with disease symptoms in each year.

| Biotype ID | Resistance | Latitude | Longitude | Habitat Type | Expmt A Percent Diseased (N) | Expmt B Percent Diseased (N) |
| --- | --- | --- | --- | --- | --- | --- |
| N4 | S | 41.76969 | -94.46573 | Non-Agricultural | 77.6 (49) | 94.0 (50) |
| N5 | S | 41.78945 | -94.31520 | Non-Agricultural | 8.0 (50) | 81.6 (49) |
| N18 | S | 40.71030 | -94.07713 | Non-Agricultural | 20.4 (49) | 81.3 (48) |
| N2 | S | 41.29313 | -94.45269 | Non-Agricultural | 20.0 (50) | 62.5 (48) |
| S26 | S | 40.75121 | -93.65490 | Soybean Field | 41.7 (48) | 93.3 (45) |
| S20 | S | 41.24791 | -93.38431 | Soybean Field | 90.0 (50) | 94.0 (50) |
| N11 | S | 41.40064 | -93.28567 | Non-Agricultural | 76.0 (50) | 100.0 (50) |
| N24 | S | 40.73947 | -93.83659 | Non-Agricultural | 78.0 (50) | 98.0 (49) |
| N37 | S | 41.01701 | -91.98267 | Non-Agricultural | 87.8 (49) | 93.8 (48) |
| S32 | LR | 41.50633 | -95.74919 | Soybean Field | 14.3 (49) | 33.3 (33) |
| N13 | LR | 40.74718 | -93.11628 | Non-Agricultural | 57.1 (49) | 90.0 (40) |
| S24 | LR | 40.71617 | -94.22263 | Soybean Field | 32.0 (50) | 83.7 (49) |
| S31 | LR | 41.60016 | -93.46246 | Soybean Field | 50.0 (50) | 84.0 (50) |
| S4 | LR | 41.74592 | -92.66809 | Soybean Field | 22.9 (48) | 52.1 (48) |
| S22 | LR | 41.41784 | -93.43370 | Soybean Field | 30.8 (39) | 85.7 (49) |
| N36 | LR | 41.84368 | -94.64285 | Non-Agricultural | 24.5 (49) | 37.5 (32) |
| S6 | LR | 41.92105 | -94.36237 | Soybean Field | 16.0 (50) | 54.2 (48) |
| S14 | ER | 40.74769 | -92.35134 | Soybean Field | 32.0 (50) | 90.2 (50) |
| S15 | ER | 40.84683 | -92.09708 | Soybean Field | 32.7 (49) | 80.0 (50) |
| S19 | ER | 41.76350 | -91.90863 | Soybean Field | 20.4 (49) | 74.0 (50) |
| S45 | ER | 41.19199 | -91.52598 | Soybean Field | 14.0 (50) | 56.9 (50) |
| S17 | ER | 40.74837 | -92.20936 | Soybean Field | 10.0 (50) | 75.0 (48) |
| N28 | ER | 40.94598 | -92.63812 | Non-Agricultural | 16.0 (50) | 88.0 (50) |
| S25 | ER | 40.85530 | -93.47446 | Soybean Field | 27.3 (44) | 92.0 (50) |
| S11 | ER | 40.76117 | -93.49468 | Soybean Field | 41.7 (48) | 72.9 (48) |
| S10 | ER | 41.10143 | -92.77837 | Soybean Field | 36.7 (49) | 46.9 (49) |

Note: Biotypes beginning with “S” are from agricultural habitats, and biotypes beginning with “N” are from non-agricultural habitats. Resistance categories are denoted as S for susceptible, LR for low-level resistant, and ER for extremely resistant (see text). Disease data are from Experiment A (2016) and Experiment B (2017); data from both sites in each year are combined with sample size in parentheses.
